# Novel Strategy to Fabricate PLA/Au Nanocomposites as an Efficient Drug Carrier for Human Leukemia Cells in Vitro

**DOI:** 10.1007/s11671-010-9762-3

**Published:** 2010-09-14

**Authors:** Jingyuan Li, Chen Chen, Xuemei Wang, Zhongze Gu, Baoan Chen

**Affiliations:** 1 State Key Lab of Bioelectronics (Chien-Shiung WU Laboratory), Southeast University, 210096 Nanjing, China; 2 Department of Hematology, Zhongda Hospital, Southeast University, 210096 Nanjing, China

**Keywords:** Human leukemia, PLA/Au nanocomposites, Daunorubicin, Drug delivery, Multidrug resistance

## Abstract

Poly (lactic acid) (PLA) polymer has the promising applications in the biomedical field because of its biodegradability and safe elimination. In this study, we have explored the bio-application of new nanocomposites composed with PLA nanofibers and Au nanoparticles as the potential drug carrier for an efficient drug delivery in target cancer cells. The results demonstrated that the anticancer drug daunorubicin could be efficiently self-assembled on the surface of PLA/Au nanocomposites and the synergistic enhancement of PLA/Au nanocomposites conjugated with daunorubicin into drug-sensitive K562 and drug-resistant leukemia K562/AO2 cells could be obviously observed by MTT assay and confocal fluorescence microscopy studies. These observations suggest that the new nanocomposites could readily induce daunorubicin to accumulate and uptake in target leukemia cells and increase the drug's cytotoxicity. Especially, the PLA/Au nanocomposites could significantly facilitate the cellular drug absorbtion of daunorubicin into drug-resistant K562/AO2 cells and efficiently inhibit the cancer cell proliferation. This raised the possibility to utilize the PLA/Au nanocomposites as a new effective additive agent to inhibit the drug resistance and thus as a novel strategy to sensitively track the respective cancer cells.

## Introduction

As one of the difficult treated diseases, cancer threats the life of many patients. To reduce the morbidity and mortality of cancer, early diagnosis and cancer systemic therapies are of paramount importance. The cure efficiency of cancer chemotherapy depends not only on the anticancer drug itself but also on how the drug reagent is efficiently delivered to its targets [1]. Although there is much effort to solve the relevant problems, it is still a big challenge to develop a new strategy to mark and track the target cancer cells for the early diagnosis and cure of cancers. Besides, the emergence of drug resistance is a worldwide puzzle in the related diseases' therapies, while the occurrence of the multidrug resistance (MDR) phenomenon is one of the major obstacles to the success of the tumors' chemotherapy [2,3]. It is noted that the mechanisms involved in drug resistance of cancer cells are pertaining to multifactor processes. And the cellular uptake of some drugs may be poor by the mutated tumor cells. The proteins related with drug resistance may pump out the drug molecules from the mutated tumor cells, which will decrease the drug concentration inside the tumor cells [4]. Thus, the efficient targeting of drug delivery for relevant cancer cells could afford a new strategy for the effective treatment of targeted cancers [5,6].

Recently, some reports have demonstrated that anticancer drugs could be readily modified on the biocompatible nanomaterials covalently or non-covalently, which could afford the sustained drug delivery for the target cancer cell lines and reduce the relevant toxicity toward normal cells and tissues [7-9]. For instance, some semiconductor nanoparticles such as TiO_2_ nanoparticles can penetrate across barriers into cancer cells to allow efficient drug accumulation at the targeted locations, which could have promising application in biomedical and bioengineering fields due to its oxidizing and biocompatible properties, chemical inertness and photoactivity [10-16]. Au nanoparticles were also applied as a potential carrier or protective container for biologically active agents [8]. With the characteristics of biocompatibility, biodegradability and absorbability, some polymers have been widely used in medical research such as DNA binding delivery with PLA/PEG nanoparticles, poorly soluble Ethaselen's delivery with mPEG-PLA copolymers, prostheses for tissue replacements, supporting surgical operation and artificial organs for temporary or permanent assistance [17-19]. Some biocompatible polymer can also act as drug carriers by controlling the release rate of the loaded drug [20-23]. Meanwhile, the blends of the biodegradable polymers have been explored for the potential applications in biomedical field such as the drug release/implants for orthopedic surgery or blood vessels due to their good biocompatibility, low cost, safe elimination, lightweight and high performance [24-29]. Thus, on the basis of these observations, the biodegradable poly(lactic acid) (PLA) nanofibers have been fabricated in this study by using electrospinning and then adopted to blend with Au nanoparticles to form a new nanocomposites with the good biocompatibility. Afterward, the PLA/Au nanocomposites were further conjugated with anticancer drug daunorubicin to efficiently facilitate the intracellular accumulation of the anticancer agents inside drug-sensitive and drug-resistant leukemia cells.

## Experimental Section

### Reagents

Daunorubicin was purchased from Nanjing Pharmacy Factory (analytical grade) and freshly prepared with phosphate buffer solution (PBS, 0.1 M, pH 7.2). Au nanoparticles were freshly prepared according to the previous report [30], in which 100 mL of 0.01% HAuCl4 was heated to boiling with uninterruptedly agitating and 3.0 mL of 1% trisodium citrate was dropped into the above solution. The system was continually agitated about 30 min until the reaction color did not change. Ultrapure water was added in which the final volume was 100 mL and the diameter of Au nanoparticle was about 15 nm. The other reagents were analytical grade. For the following studies, all experimental measurements were performed at least three times in parallel.

### The Preparation of Poly(lactic acid) Nanofibers

Poly(lactic acid) nanofibers were fabricated by electrospinning. First, the poly(lactic acid) (Mn = 340,000) was dissolved in the solvent of chloroform (10%, wt) and stirred for 2 h. Next, it was loaded into a syringe connected with anodal voltage. Aluminum foil as a collecting substrate was connected with cathodal voltage. Electrospinning was performed at room temperature with a gap between the substrate electrode and the tip of the capillary of 10 cm at driving voltages of 10 kV. The PLA nanofibers were characterized by scanning electron microscopy (Magnification: 10,000) as shown in our previous report [31].

### The Preparation of PLA/Au Nanocomposites Conjugated with Dauborubicin

The aqueous suspension of PLA nanofibers was prepared in double-distilled water by ultrasonic treatment for about 20 min. Then, Au (2.80 × 10^-7^ mol/L) and PLA nanofiber gel aqueous solutions (1.3 × 10^-3^ g/L) were mixed and incubated together for more than 12 h to form the PLA/Au nanocomposites. The solution of daunorubicin (3.3 × 10^-4^ mol/L) was mixed into the nanocomposites and stored in the dark at 4°C for more than 12 h to form the PLA/Au nanocomposites conjugated with dauborubicin.

### Atomic Force Microscopy Study

In a relevant atomic force microscopy study (AFM), 5 μL from the aforementioned different solutions was deposited onto freshly cleaved mica (already glued on a steel disk) and incubated for 5 min. After the sample dried under a nitrogen stream, imaging was performed in tapping mode, by using a Nanoscope IIIa Multimode AFM (Digital Instruments, Santa Barbara, CA, USA) operating in air at room temperature.

### MTT Assay

For cell culture, human leukemia cells (K562 and K562/AO2) were cultured in a flask in an RPMI 1640 medium (GIBCO) supplemented with 10% fetal calf serum (FCS, Sigma), penicillin (100 mU/mL) and streptomycin (100 μg/mL) at 37°C in a humidified atmosphere containing 5% CO_2_, while 1 μg/mL doxorubicin was contained in the culture to maintain K562/AO2 cells' drug resistance in its daily culture.

The inhibition of cell growth was measured by MTT (Microculture Tetrazolium) assay. Initially, the respective leukemia cells (K562 and K562/AO2) in the log phase were seeded in a 96-well plate at a concentration of 1.0 × 10^4^ cells) well. The target cells were treated with different concentration of PLA/Au nanocomposites, daunorubicin or daunorubicin conjugated with PLA/Au nanocomposites, respectively. Controls were cultivated with the respective solvent under the same conditions. Each culture was incubated for 48 h in a 5% CO_2_ incubators at 37°C, and then 20 μL of 5 mg/mL MTT was added to the wells and incubated for an additional 4 h. Subsequently, it was centrifuged at 1,000 rpm for 10 min and the supernatant was discarded, followed by the addition of 150 μL of dimethyl sulfoxide (DMSO) into each well and then incubated in the shaker at 37°C with gentle shaking about 5 min. Then, the optical density (OD) was read at a wavelength of 492 nm.

### Laser Confocal Fluorescence Microscopy

The cell culture conditions were similar to those described above. Initially, the different cells were collected by centrifugation at 1,000 *g* for 5 min. Then, the supernatant solutions were discarded. The pellets were resuspended with PBS to eliminate the effect of medium in the fluorescence detection. And the cell suspension was detected on a Leica TCS SP2 (Leica). In the control experiments, only daunorubicin or PLA/Au nanocomposites were injected for relevant cellular incubation. The freshly prepared cell culture was dropped on a strictly cleaned glass plate immediately before the measurement. The excitation wavelength of fluorescence was 480 nm. All the optical measurements were carried out at room temperature (20 ± 2°C).

## Results and Discussion

### AFM Study of PLA/Au Nanocomposites Conjugated with Daunorubicin

**Figure 1:**
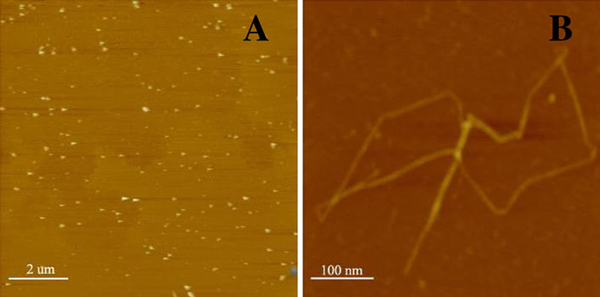
**Typical AFM images of a Au nanoparticles, and b PLA nanofiber**.

**Figure 2:**
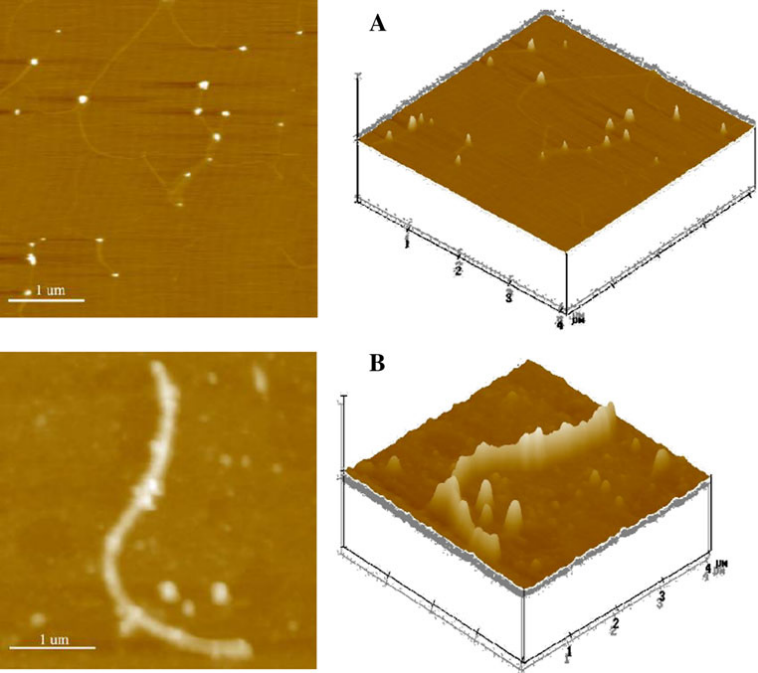
**Typical AFM images of nano PLA/Au polymer nanofibers (a) and PLA/Au nanocomposites conjugated with daunorubicin (3.30 × 10^-4^ M) (b)**. *Z Scale*: 200 nm.

As shown in Figures 1 and 2, the AFM images of PLA nanofibers, Au nanoparticles and the blending of the PLA/Au nanocomposites with daunorubicin demonstrate that upon blending of daunorubicin with the PLA/Au nanocomposites, some relatively large nanospheres appear at PLA chains. It is observed that there was a discernible substrate under the spherical particles, which is at the same level as the pure PLA nanofiber, the average height of which is about 1.5 ± 0.05 nm. Meanwhile, relevant measurements show that the average height of the conjugated nanocomplexes was (26 ± 0.62) nm, and the average outer diameter of spherical nanoparticles was (22 ± 0.55) nm. It is apparent that the Au nanoparticles and the drug molecules could self-assemble or pack together to form the spherical particles on poly(lactic acid) nanofibers, as shown in Figure 2. The rational behind this could be attributed to the fact that daunorubicin is positively charged while the relative surface of nano PLA/Au polymer nanofibers is negatively charged in pH 7.2 PBS solution. Thus, daunorubicin could be readily self-assembled onto the surface of PLA/Au nanocomposites through electrostatic interaction and other non-covalent binding.

Since the PLA nanofiber has a very high continuous surface area, it has attracted a great deal of attention in fabricating continuous ultrafine fibers or fibrous structures for various polymers, with typical examples including engineering plastics, biopolymers and polymer blends [32,33]. From the specific nanostructure of the PLA nanofibers and the relevant nanocomposites observed in above AFM study, it is evident that the anticancer drug daunorubicin could be readily self-assembled on the surface of the new PLA/Au nanocomposites, which could be utilized as a new promising carrier for nanomedicine in cancer treatment.

### Fluorescence Imaging of Intracellular Drug Delivery in Leukemia Cancer Cells

**Figure 3:**
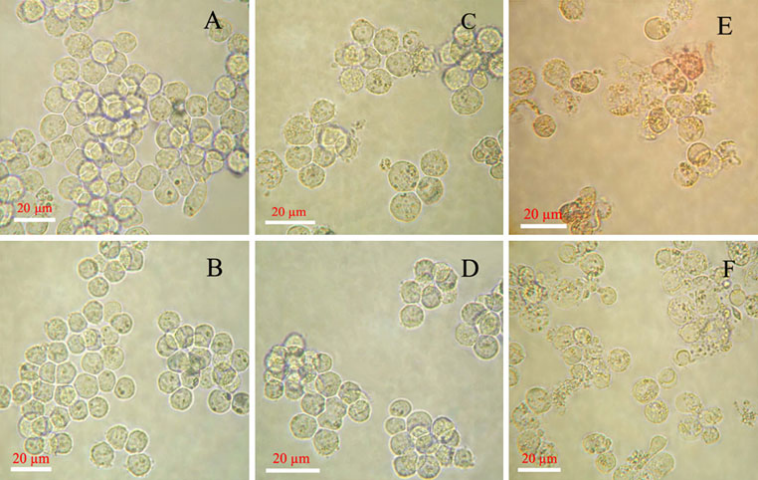
**Optical microscopy images of leukemia cancer cells**. **a** K562 cells, **c** K562 treated with DNR, **e** K562 treated with DNR conjugated with PLA/Au nanocomposites (DNR was 1 × 10^-6^ M in the above systems); **b** K562/AO2 cells, **d** K562/AO2 treated with DNR, **f** K562/AO2 treated with DNR conjugated with PLA/Au nanocomposites (DNR was 1 × 10^-6^ M in the above systems).

Based on the above observations, the PLA/Au nanocomposites have been further explored as a new potential drug carrier for efficient drug delivery. Initially, the microscopy images of leukemia cancer cells in the absence and presence of PLA/Au nanocomposites have been investigated by optical microscopy. As shown in Figure 3, it is observed that the drug-sensitive leukemia cancer cells K562 and drug-resistant leukemia cancer cells K562/AO2 had the good morphology in the negative control. While K562 cells were cultured with DNR conjugated with PLA/Au nanocomposites, significant morphological changes were detected and more cell death occurred than that of cells treated with DNR alone. In comparison, there were no any morphological changes for drug-resistant leukemia cells K562/AO2 after treated with DNR alone because of the relevant multidrug resistance, as shown in Figure 3b and 3d. After the cells were treated by DNR conjugated with PLA/Au nanocomposites, significant increase in the cell death could be detected. Considering the good biocompatibility of PLA and Au nanomaterials, these observations suggest that the apparent increase in cancer cell death should be attributed to the synergistic function derived from the combination of DNR with PLA/Au nanocomposites. Especially, the PLA/Au nanocomposites–DNR complexes can remarkably facilitate the accumulation of the DNR molecules in the drug-resistant cancer cells and apparently reserve the MDR of K562/AO2.

**Figure 4:**
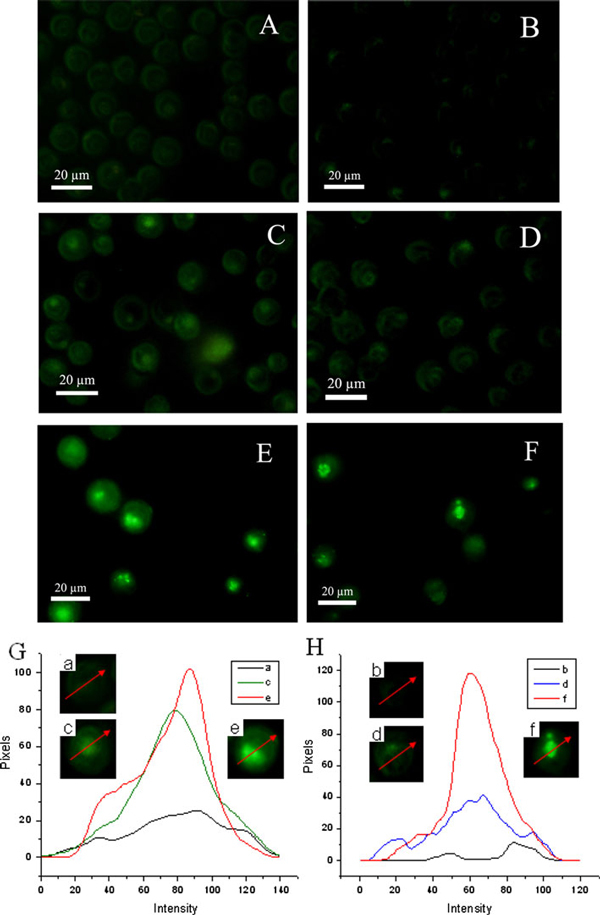
**Confocal fluorescence microscopy images of different leukemia cancer cells treated with anticancer agents**. **a** K562 treated with DNR, **c** K562 treated with DNR and PLA nanopolymers, **e** K562 treated with DNR conjugated with PLA/Au nanocomposites (DNR was 1 × 10^-6^ M in the above systems); **b** K562/AO2 treated with DNR, **d** K562/AO2 treated with DNR and PLA nanopolymers, **f** K562/AO2 treated with DNR conjugated with PLA/Au nanocomposites (DNR was 1 × 10^-6^ M in the above systems). **g** and **h,** respectively, give the quantitative fluorescence intensity curves of images for K562 system (**g**) and K562/AO2 system (**h**) in which the single cell was respectively selected from the above related systems.

With the good fluorescence characteristics, DNR could also be utilized as the fluorescence probe to track its location and concentration inside the cells. As shown in Figure 4, the synergistic effect for the uptake of DNR on K562 and K562/AO2 could be obviously observed by the laser confocal fluorescence microscopy. Figure 4 showed the typical images of the confocal fluorescence microscopy of different leukemia cancer cells. It appeared that the relatively weak drug uptake was observed when the cancer cells were only treated with DNR. While the intracellular fluorescence was slightly strengthened after relevant cells incubated by DNR together with PLA nanofibers. In comparison, when PLA/Au nanocomposites conjugated with DNR were incorporated into the target system, PLA/Au nanocomposites have an apparent synergistic effect on the drug uptake of DNR in the respective cancer cells, where the intracellular fluorescence intensity was remarkably enhanced upon application of the PLA/Au nanocomposites together with DNR, as shown in Figure 4e and 4f. Since the PLA/Au nanocomposites themselves have no fluorescence, the intracellular fluorescence was only generated by the anticancer drug DNR. Our previous study indicates that the presence of bare Au nanoparticles alone could just slightly enhance the uptake of DNR by K562 cells [8]. Hence, these results indicated that much more DNR molecules could be efficiently accumulated in the cancer cells upon application of DNR together with PLA/Au nanocomposites.

Additionally, our observations demonstrate that for drug-resistant K52/AO2 cells treated with DNR alone, scarce cancer cells with weak intracellular fluorescence could be observed. The very weak intracellular fluorescence of drug-resistant leukemia cells treated with DNR alone may be attributed to the over-expression of P-gp protein on the cell membrane of the drug-resistant cancer cells [34,35], which could readily pump DNR molecules out of the relevant cancer cells so that much lower intracellular DNR fluorescence could be observed for K562/AO2 cells than that of K562 cells, as shown in Figure 4a and 4b. Interestingly, the presence of PLA/Au nanocomposites could efficiently facilitate the drug uptake of DNR into the drug-resistant leukemia cells and lead to the much higher intracellular drug concentration in the target cells, resulting in the remarkable enhancement of the intracellular fluorescence of drug-resistant leukemia cells (Shown in Figure 4b and 4f). This result suggests that the PLA/Au nanocomposites may affect the activity of P-gp protein and efficiently prevent the drug efflux from the drug-resistant leukemia cells. Thus, much more DNR could be readily permeated and accumulated into the relative cancer cells because the relevant synergistic effect of the PLA/Au nanocomposites could apparently inhibit the drug resistance of K562/AO2. In view of these observations, it appears that the PLA/Au nanocomposites may play as potential inhibitor of multidrug resistance (MRD) and thus efficiently promote the cellular uptake of the drug into the relevant drug-resistant cancer cells.

### Cytotoxicity Study by MTT Assay

**Figure 5:**
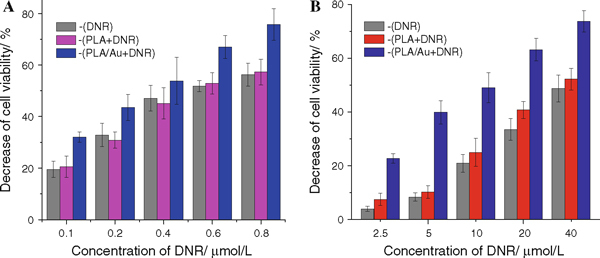
**MTT assay to test the cytotoxicity of DNR in the presence of PLA or PLA/Au nanocomposites for K562 cells (a) and K562/AO2 cells (b) for 48 h**. *Error bars*, ±SEM.

The cell viability of leukemia cancer cells in the presence of PLA/Au nanocomposites loaded with DNR has been explored by MTT assay. As shown in Figure 5, the results demonstrate that the combination of the PLA/Au nanocomposites with DNR could more effectively inhibit the growth of these two different kinds of leukemia cells than that treated with DNR alone. It is evident that the biocompatible PLA/Au nanocomposites have a synergistic effect to facilitate the drug uptake into human leukemia cells, increase the relative intracellular drug concentration and hence enhance the cytotoxicity of anticancer agents. Meanwhile, it is observed that the inhibition effect for drug-resistant K562/AO2 cancer cells was relatively significant than that for drug-sensitive K562 cancer cells when treated with DNR conjugated with PLA/Au nanocomposites. As shown in Table 1, our results revealed that the resistant factor of the reversal index to resistant leukemia cells was 70.14 for K562/A02 in the control group, while the resistant factor in the presence of the PLA/Au nanocomposites significantly decreased to 38.88; therefore, the reversal index to K562/A02 was 1.8. This suggests that the presence of PLA/Au nanocomposites can reinforce the accumulation of DNR in drug-resistant K562/A02 cells and lead to a great extent decreasing of the resisting factors. Thus, the interaction of PLA/Au nanocomposites with bioactive molecules on the cell membrane could provide a new strategy to overcome the multidrug resistance (MDR) of K562/AO2 cells by improving the efficiency of drug delivery. These observations were coherent with the above results of confocal fluorescence studies.

**Table 1:** The resistant factors and reversal index of leukemia K562 and K562/AO2 cell lines to PLA/Au nanocomposites

Groups	IC50/μmol/L		Resistant factors	Reversal index
			
	K562	K562/AO2		
Control group	0.51	35.77	70.14	1.8
Experimental (PLA/Au) group	0.25	9.72	38.88	

## Conclusion

In summary, in this study we have fabricated the new PLA/Au nanocomposites and explored the promising application of the PLA/Au nanocomposites to efficiently facilitate the uptake of anticancer drug in target cancer cells. Our observations demonstrate that the self-assembly and conjugation of anticancer drug DNR on the surface of PLA/Au nanocomposites could significantly enhance the drug accumulation into drug-sensitive K562 and drug-resistant leukemia K562/AO2 cells and thus increase the drug's cytotoxicity. Importantly, the PLA/Au nanocomposites could considerably reverse the multidrug resistance of K562/AO2 cells and efficiently inhibit the cancer cell proliferation. This raised the possibility to utilize the PLA/Au nanocomposites as a new effective additive agent to overcome the drug resistance and thus as a novel strategy to sensitively track the respective cancer cells for efficient cancer chemotherapy.
